# Legislation has Changed But Issues Remain: Provider Perceptions of Caring for People Who Use Cannabis During Pregnancy in Safety Net Health Settings, a Qualitative Pilot Study

**DOI:** 10.1089/whr.2023.0057

**Published:** 2023-07-28

**Authors:** Rachel Carmen Ceasar, Erin Gould, Julia Stal, Jen Laughter, Michelle Tran, Shirlene D. Wang, Jordan Granacki, Ryan S. Ziltzer, Jasmeen Joy Santos

**Affiliations:** ^1^ Department of Population and Public Health Sciences, Keck School of Medicine, University of Southern California, Los Angeles, California, USA.; ^2^ Department of Sociology, California State University, Fullerton, Fullerton, California, USA.

**Keywords:** cannabis, pregnancy, prenatal cannabis use, health care provider knowledge, maternal health, substance use

## Abstract

**Objective:**

To identify perceptions of cannabis use and risk among maternal health providers who provide care for people who use cannabis during pregnancy in safety-net health settings.

**Methods:**

Using qualitative, constructivist ground theory methods, we conducted semistructured remote interviews with 10 providers (2 midwives, 6 OB/GYN physicians, and 2 OB/GYN residents) in Southern California, United States, between March 15, 2022, and April 6, 2022. We selected participants through selective sampling using a convenience sample and snowball approach. Providers were eligible for the study if they self-reported *via* survey to being a maternal health provider (*e.g*., physician, doula, midwife, and so on) providing care in a safety-net health setting and had cared for people who used cannabis during pregnancy in the last year. Analysis drew upon grounded theory methods to document the socio-structural contexts that contribute to provider perceptions about cannabis. This study was approved by the University of Southern California Institutional Review Board (UP-21-00282-AM009).

**Results:**

We identified three categories of provider perceptions of cannabis use and risk during pregnancy: (1) Relying on self-education, (2) Taking a case-by-case approach, and (3) Avoiding cannabis discussions to maintain an alliance with patients. Findings indicate that provider reluctance to counsel patients about cannabis in favor of preserving a therapeutic relationship can overlook the lack of resources and access to health care alternatives available to low-income patients that can shape self-medicating.

**Conclusions:**

Nonpunitive policies and training on cannabis use are critical steps for supporting providers to counsel patients who use cannabis during pregnancy, alongside a harm reduction approach that acknowledges the broader socio-structural contexts and barriers facing patients who disclose use.

## Introduction

Cannabis is the most commonly used illicit substance in pregnancy in the United States.^1,2^ People are increasingly using cannabis for its perceived health benefits, such as to self-treat physical and psychological issues related to pregnancy (*e.g*., nausea, pain, depression, anxiety, coping).^3,4^ While cannabis may have some therapeutic benefits,^5^ the American College of Obstetricians and Gynecologists (ACOG)^6^ and the American Academy of Pediatrics (AAP)^7^ recommend that providers counsel pregnant patients to abstain from use due to concerns that cannabis could interfere with fetal neurodevelopment. Yet the effects of cannabis during pregnancy have been inconsistent and unclear due to confounding variables and lack of consistent measures.^8,9^

Without definitive evidence demonstrating the exact adverse outcomes and magnitude of risks, there is often confusion and miscommunication among pregnant people and providers about the use of cannabis during pregnancy. Research shows that providers are hesitant to discuss cannabis use with their pregnant patients due to insufficient evidence on the effects of cannabis use during pregnancy and a lack of confidence in counseling about use.^10^ When patients do disclose their cannabis use during pregnancy to providers, they report a lack of adequate counseling and dissatisfaction with the quality of information they receive.^11^

As states continue to legalize cannabis use, providers will play a critical role in guiding patients about cannabis use during pregnancy. For example, in a recent systematic review of clinician counseling approaches, 2 of 13 studies noted a punitive counseling style where clinicians threatened patients with the involvement of child protective services to encourage cessation of cannabis use.^12^ While the socio-legal implications of provider approaches on cannabis use during pregnancy have been well studied, research examining provider perspectives within a broader health inequity framework is limited.^13^ Understanding the unintended maternal health consequences of legalization and the role providers play in mitigating them is critical to minimize barriers to perinatal care for pregnant people, especially for those with limited resources who are accessing care in safety net health settings.

As punitive state laws related to pregnancy and substance use have proliferated and policies are applied that target low-income and Black, Indigenous, and People of Color (BIPOC) people,^14–19^ it is critical to study how social-structural contexts—such as provider bias or opinion—play a role in provider decision-making surrounding patient cannabis use during pregnancy. In this study, we draw upon findings from qualitative interviews with maternal health providers who care for people who use cannabis during pregnancy in safety net health settings (*i.e*., health settings that primarily serve uninsured, Medicaid-insured, and other vulnerable populations),^20^ in Southern California to examine their perceptions of cannabis use and risk. The primary objective of this pilot study was to gather preliminary data on provider perceptions of cannabis use and their approaches to caring for patients who use substances during pregnancy and how this approach may contribute to maternal health inequities.

## Methods

### Study design—constructivist ground theory

This pilot study is part of a broader qualitative pilot project that included interviews with providers, as well as people who use cannabis during pregnancy.^21^ The research team consisted of advanced public health students enrolled in a 16-week graduate-level qualitative methods seminar led by the Principal Investigator of the study. We reviewed COREQ, a 32-item checklist, throughout the study, to support the reporting of important aspects of our research team, methodology, findings, and analysis (Supplementary Appendix SA3: COREQ checklist).^22^ We conducted a qualitative study using constructivist grounded theory to understand socially constructed concepts of risk and safety surrounding cannabis use, the phenomenon under study.

### Selection of participants—sampling and recruitment

We selected participants through selective sampling using a convenience sample and snowball approach to generate an information-rich group of maternal health providers who practice in Southern California and have experience in caring for people who use cannabis during pregnancy. This type of nonrandom sampling was based on team discussions about selecting participants that would provide insightful information regarding the research question.^23,24^ Providers were eligible for the study if they: (1) self-reported that they were a provider (*e.g*., physicians, doula, midwife), (2) provided care to patients in safety net health settings in Southern California, and (3) cared for people who used cannabis during pregnancy in the last year. People who responded to a request to be part of the study were emailed a HIPAA-compliant REDCap survey to confirm their eligibility, sign an e-consent form, and scheduled a HIPAA-compliant Zoom interview.

### Data collection—semistructured interviews

The semistructured interview guide drew upon existing qualitative and quantitative literature (Supplementary Appendix SA1: Interview guide).^3,11,12,25^ We piloted and refined the interview guide within the research team, which was made up of students with clinical and/or public health knowledge. We revised the interview guide as the interviews progressed to refine questions and pursue areas identified as theoretically relevant. The refining of the interview guide was part of an iterative process aimed at generating richer responses from participants.

The 60-minute interview occurred remotely *via* HIPAA-compliant Zoom from March 15, 2022, to April 6, 2022. Each interview was conducted by two or more people—one person led the interview session, while a second person focused on follow-up questions and analytical note-taking. We followed up on questions with open-ended inquiries about topics introduced by the participant (*e.g*., lack of mental health services for pregnant people). This nondirective, open-ended approach of qualitative interviewing encourages participants to elaborate beyond the original scope of the interview guide and allows for perspectives that are not anticipated from the scientific literature or by the research team.^26^

Once an interview was completed, we sent audio recordings to an external transcriber for transcription. We then uploaded and stored the deidentified transcripts in a HIPAA-compliant OneDrive server, and audio and/or video files containing identifiers were deleted. Throughout the data collection phase, the team wrote summaries of each transcript and memos that would detail emerging insights and patterns as part of their preliminary analysis. Data collection proceeded past the point of theoretical saturation, meaning that insights and patterns emerging from additional interviews aligned with existing data characteristics.^27^

### Data analysis—grounded theory methods

We used a constructivist grounded theory methodology to understand how a group of people (providers) construct meaning around an area of inquiry (cannabis use during pregnancy).^28^ We sought to develop a conceptual framework (theory) through analyses and constant comparisons across the data.^27^ This method is best used when little is known about a phenomenon, with the aim of generating an explanation or theory from the data based on participants' narratives and experiences.

Once the final interview was completed, transcribed, and memoed, we reviewed and categorized the initial memos of all the transcripts into emerging subject areas that formed the basis of a codebook. For our first draft of the codebook, we developed definitions and wrote out examples from the transcripts for each code, which captured actions and underlying ideas shared by participants that were indicative of a larger framework of meaning. Each team member wrote a draft codebook based on their own insights from the transcripts which were then pooled together and discussed as a team to create a final draft. The team consolidated codes that overlapped, deleted codes that were not representative of the data, considered alternatives, and modified codes to reflect the data more accurately and refine ideas. This was done until consensus was achieved on which codes were the best explanatory themes of the data. We tested the codebook by applying and comparing it to multiple transcripts to ensure accurate analysis and then made revisions before arriving at a final set of 16 thematic codes (Supplementary Appendix SA2: Codebook).

The remaining transcripts were independently coded by two different team members to ensure the consistent application of codes using ATLAS.ti software. Final memos that emerged during the coding process were shared to generate a deeper discussion of patterns and themes across all data points as categorized using the codebook. We developed these memos into new concepts (*i.e*., major categories or theories)^29^ because they reflect overlapping, frequently occurring codes, which indicated conceptual frameworks warranting deeper analysis. These broad findings of our research became the three results of this article which aim to explain the perceptions and behavior of maternal health care providers regarding perinatal cannabis use.

## Results

We interviewed 10 providers who self-reported providing care in safety net health settings for people who use cannabis during pregnancy in Southern California (Table 1). While participants performed a variety of specializations, 80% were physicians working in OB/GYN settings. Two participants (20%) were certified nurse midwives and eight participants (80%) were physicians, including two residents. Mirroring national averages,^30^ White individuals (70%) and people who used she/her pronouns (60%) were overrepresented among participants, with only one participant identifying as Black/African American (10%) and one person as Mixed race (10%).

**Table 1.** Maternal Health Provider Characteristics

	*n* (%)
Maternal health provider, cares for BIPOC pregnant people	10 (100)
Cares for	
People who use cannabis during pregnancy	10 (100)
People who use alcohol or other substances during pregnancy	9 (90)
People who use cannabis after pregnancy	10 (100)
Maternal health role:	
Doula/lactation consultant	1 (10)
Certified nurse midwife	1 (10)
Physician	8 (80)
Specialty	
OB/GYN	9 (90)
Labor and delivery	2 (20)
Postpartum	2 (20)
Community health	1 (10)
Midwifery	1 (10)
Doula	1 (10)
Lactation consultant	1 (10)
Maternal health provider's racial identity	
Black or African American	2 (20)
White	7 (70)
Mixed race	1 (10)
Maternal Health Provider's Pronouns	
He/him	2 (20)
She/her	7 (70)
Declined to disclose	1 (10)
Maternal health provider's age	
Under 30	2 (20)
30–39	3 (30)
40–49	3 (30)
50–59	2 (20)

BIPOC, Black, Indigenous, and People of Color.

### Result 1: With limited training, providers relied on self-directed education on cannabis to counsel patients

Providers described having little medical training surrounding cannabis use during pregnancy. As a result, many felt they needed to pursue their own self-directed education on cannabis if they wished to be adequately informed:

“It really is on you to read about it…Is there data on whether edibles are better than smoking weed? I don't know…Unfortunately, I think there could be a better, more structured education about substance use in general in pregnancy.” (Provider-2)

When providers did undertake their own research on cannabis, they found that the data were “conflicting” regarding risk:

“[T]here's a scare tactic of ‘marijuana use causes perinatal death’ and the actual data shows that that's not true…[S]tudies that show higher risk of still birth, but those are confounded by cigarette smoking and these other factors…it's conflicting.” (Provider-5)

Providers indicated that they received minimal training on this topic despite the frequency at which they encountered patients who used cannabis during pregnancy. For example, a third of participants commented that while cannabis use during pregnancy seemed just as common as gestational diabetes, they received little training on screening or managing cannabis use:

“It's probably as common as diabetes in pregnancy, and we get a ton of education about diabetes in pregnancy…” (Provider-3)

Providers felt that cannabis use was not being prioritized as a maternal health issue compared to diabetes:

“Are we definitely counseling our patients to quit? Yeah, for sure. But are we as worried about that as their uncontrolled diabetes? Probably not.” (Provider-8)

Of the little training that providers did receive on cannabis, it was largely limited to an addiction framework with little information on perinatal cannabis use and its effects:

“[Cannabis] tends to be touched on in maybe a psych or mental health block [a multi-week course focused on immersion in one topic], as a substance use disorder that you can describe in the DSM-5 [Diagnostic and Statistical Manual of Mental Disorders, Fifth Edition]. Beyond that, I don't know if we learn much about long-term health effects of cannabis use in any form. I don't know if we learn a lot about effects on pregnancy, really.” (Provider-8)

Beyond medical school, one provider noted how an addiction framework also informed their knowledge of cannabis use:

“We had [a high-risk obstetric gynecologist] come give her presentation…She runs the substance use disorder clinic [for pregnant people].” (Provider-7)

### Result 2: Without clinical guidelines, providers weighed cannabis pros and cons on a case-by-case basis in the face of limited alternative resources available to patients

Providers shared a broad range of approaches to cannabis, but in weighing the risks and benefits, they generally advised patients to consider the risks due to a lack of evidence on the effectiveness of cannabis during pregnancy:

“[I]f you're looking for hard advice from me, I'm always gonna tell you not to use any substance in pregnancy.” (Provider-8)

Providers compared the risks of cannabis to other substances, such as alcohol and tobacco. Some felt that cannabis use had no place in pregnancy and did not believe that it had any therapeutic value:

“We've legalized an evil thing and it's very hard to message to pregnant women, ‘Don't use this evil thing that we're advertising, that everybody's using casually,’ that is now felt to be safer than alcohol…Don't even get me started on vaping…I don't think any of us take marijuana use in pregnancy casually in the sense that it's okay if you do it. And I don't think any of us think it helps with nausea. That's just garbage.” (Provider-7)

Other providers shared how they struggled to juggle both the perceived therapeutic benefits and risks of cannabis use for both mother and child during pregnancy:

“Anxiety, depression, nausea…[Cannabis] certainly has its place, in my opinion…Pregnancy is a difficult one for me, because we have to advocate for the baby, and that baby is sight-unseen.” (Provider-6)

Providers repeatedly commented on the lack of institutional policies regarding cannabis use in pregnancy. Providers felt that without clear clinical guidelines, their counseling on cannabis with patients was ultimately variable and predominantly influenced by their own “biases” or their personal approach to a patient's cannabis use rather than evidence-based data, which were limited:

“[O]verall, there are biases that doctors have, that they bring into patient encounters.” (Provider-5)

“ACOG is really good at leaving it up to the OB/GYN. When there's not a lot of data, a lot of the recommendations are like…‘Individualize based on the patient.’ They don't have firm stances when there's not a lot of data, so I don't think there would be a firm stance [for providers either].” (Provider-2)

Individualized patient plans made at the discretion of the provider were a recurrent theme due to unclear guidelines at the institutional level for cannabis use in pregnancy. The participant above went on to point out the importance of decriminalizing cannabis through legalization, especially for BIPOC people, yet felt that state laws failed to consider how legalization would affect current clinical guidelines and practice:

“We know historically people of color were being incarcerated for cannabis, so from that standpoint, it's good to legalize it…[b]ut a lot of thought wasn't put into place on different policies…when it was legalized.” (Provider-2)

Without unified clinical guidelines, provider efforts to adapt a harm reduction approach to cannabis use were often made on a case-by-case basis:

“It kind of depended on the patient, honestly. If a patient had multiple substance use issues…I'm a proponent of harm reduction. I was just happy that they told me…I cannot say [cannabis] is safe, but it might be better than doing meth.” (Provider-3)

While other substances such as heroin or methamphetamine were seen as severe, providers often viewed cannabis as the safer substance to use during pregnancy and would not necessarily recommend abstaining in this case:

“If their cannabis use is preventing them from using heroin, it might be reasonable to work with them through their cannabis use disorder rather than being like, ‘You should stop right now.’” (Provider-8)

While most providers saw cannabis as a less risky substance, others felt cannabis use may suggest hidden maternal struggles for patients. One provider saw cannabis use as indicative of an individual struggling to safely take care of themselves and their baby:

“Because even though it's technically legal, it's still a marker…for folks who are on some level not doing the best they can for themselves and their babies during the pregnancy.” (Provider-7)

While some providers felt that cannabis use signaled a struggling patient, others felt that it signaled the limitations of providers and the health care system to provide resources for patients. One provider described how even if there was a strong therapeutic relationship between patient and provider, a lack of resources and access to health care might encourage cannabis self-medication in pregnancy:

“Why are you using it?…because obviously, there's probably something else going on, that I can help address. If it's anxiety, I can help you with that. If it's sleep, I can help you with that. If it's appetite issues…If we had actual resources to help, to have the time to be like, ‘Okay, let's talk about it. I can put you in touch with someone who is going to be better at helping you with this and dealing with all the stressors that you have’…We could provide better care.” (Provider-3)

Providers felt that barriers to providing optimal care to patients who use cannabis during pregnancy extended beyond a lack of protocols. One provider emphasized that solutions to such challenges required systematic change, such as the need for improvements in health literacy and psychosocial support for their patient population:

“The average reading level in…both hospitals I work at is third grade…You're not gonna fix this by handing out flyers in the prenatal clinic that say, ‘Hey, you shouldn't smoke marijuana when you're pregnant.’…People are anxious. They've been scared out of their minds for 2 years with COVID. They'll do anything to make the anxiety go away. And they think that [cannabis] will help them.” (Provider-7)

### Result 3: Providers sought to preserve the patient-provider relationship at the cost of not talking about cannabis with patients

Despite a lack of training and clinical guidelines, providers felt it was their responsibility to counsel patients about their cannabis use:

“[W]e have a responsibility to talk about it, whether it's uncomfortable or not…the onus is on us to at least have those uncomfortable conversations, so that we can try to educate our patients regarding better behaviors…” (Provider-6)

Yet many providers felt unprepared to have conversations about cannabis use with their patients. To avoid alienating patients in prenatal visits, one provider shared how they refrained from asking direct questions pertaining to cannabis use to preserve the patient-provider relationship:

“I'm juggling a desire to understand with a desire not to make people feel judged. I often will avoid asking questions about…where are you getting your cannabis, and what kind…the details of your use. Which you could argue that I should be getting [this information]…[A]nytime I'm talking about marijuana use with people, I'm nearly apologizing for talking about it, because I so desperately want to hold on to that therapeutic relationship…” (Provider-5)

Other providers also emphasized the importance of maintaining trust and rapport with patients while minimizing feelings of discomfort and stigma:

“[W]e…don't want to make these women feel like they can't tell us things, or that they are ‘bad’ for using [cannabis]…” (Provider-1)

Providers expressed apprehension regarding counseling due to concerns that a strong position on cannabis could damage the patient-provider relationship, resulting in loss of care or the patient conflating the provider's views with the criminal justice system:

“If you are hammering them on one visit, sometimes it deteriorates your therapeutic relationship…[W]e really want [patients] to come back and view us as benevolent people rather than the system. With cannabis being legalized but still stigmatized, even in California, it's still equated with criminality. And…people who advise you not to use cannabis are equated with criminal justice and that is a hard line to walk for physicians.” (Provider-8)

This same provider went on to suggest that a mutual support group among people who use cannabis during pregnancy could be one way to supplement provider discussions around cannabis use education that could be influential for patients:

“We have enough of a population of cannabis users in pregnancy that they could be in a mutual support group together to talk about cannabis use in pregnancy. The advice you receive from your physician is only one component. And if you're getting advice from peers too, that goes a long way.” (Provider-8)

## Discussion

This study builds upon research on provider perceptions of cannabis use and risk to document the socio-structural contexts that contribute to provider decision-making about cannabis use during pregnancy.^12^ Similar to prior research, our findings show that providers received little to no training on cannabis use during pregnancy,^31^ and relied on self-directed education to inform their knowledge gaps. We found that without a unified approach to counsel patients about cannabis use, providers often drew upon their own views of cannabis to inform practice. While some providers applied an addiction framework to cannabis, others took on a harm reduction approach, perceiving cannabis as less risky than other substances and an understandable option for patients accessing care in a medical system with limited resources. This is consistent with previous research in which providers reported varying stances on perinatal cannabis use with some offering supportive nonjudgmental care and others expressing greater concern for cannabis use and other substances.^31^ Coupled with a lack of knowledge about cannabis use and an awareness of the link between the medical and criminal justice systems, providers struggled to counsel patients about cannabis use and preserve the provider-patient relationship for fear of stigmatizing patients and losing their care.

Our findings suggest that without nonpunitive policies and clear clinical guidelines, providers will continue to avoid or reluctantly counsel patients on cannabis use during pregnancy. These results are consistent with prior qualitative studies on cannabis use during pregnancy which showed that providers often avoid asking patients about any substance use—be it cannabis,^10^ or tobacco or alcohol.^32^ As with cannabis, research shows that provider adherence to implementing guidelines for more commonly used substances, such as alcohol and tobacco, through the evidence-based Screening, Brief Intervention, and Referral to Treatment (SBIRT) strategy is shown to be inadequate, even when available for digital delivery.^33^ Our findings are consistent with research on other substances which shows that providers are often reticent to inquire, screen, or provide referrals for patients' substance use for fear it may interfere with the clinical relationship, despite patients reporting openness to discussing their substance use with providers.^34^

Our research also demonstrates that providers have varying views of cannabis legalization and these views may directly or indirectly impact the care they provide patients who disclose use. With expanding legalization and the potential for federal legalization in the United States, we anticipate that maternal health providers will be seeing more people using cannabis during pregnancy as prevalence and frequency of prenatal cannabis use have increased in recent years.^35,36^ Patients may also feel more comfortable disclosing perinatal cannabis use to health care providers due to increasing legality.^31^ Given this, it is imperative that maternal health providers be a reliable and nonjudgmental source of information on cannabis use during pregnancy for patients.

Prior research has called upon evidence-based public health campaigns to increase education and awareness among pregnant people about the potential risks of cannabis.^37^ But there has been less emphasis on increasing education and awareness among providers, who have a direct role in informing patients about their maternal health decisions.^38^ Our findings support prior research showing that pregnant people will seek out cannabis information from providers,^10,39–41^ but that providers are not a preferred source of information for patients because they lack clear information and patients fear punitive responses from them.^4,11,42^

It is critical for public health campaigns to build confidence in providers about how to respond to a disclosure of cannabis use. This means providing patients with clear research and evidence that addresses safety concerns and offers harm reduction options. This also means equipping providers with clear and consistent data that can supplement anecdotal information pregnant people are already gathering from friends, family,^11^ the internet, and/or cannabis retailers.^4,38^ Pregnancy is often described in public health as a “window of opportunity” for people to change their behaviors—such as substance use—or a “gateway” or introduction to bring patients with low-income into the health system.^43,44^ Yet there has been little discussion regarding how providers might leverage the pregnancy period to address the larger structural barriers facing people when accessing care (*e.g*., stigma, involvement of criminal justice system, loss of child custody, pregnancy-related mortality). Our study supports ongoing research to better understand the barriers providers face in counseling patients on cannabis use during pregnancy and inform harm reduction communication and training resources that consider the structural challenges facing patients during the perinatal period.^45^

### Limitations

This study has limitations. First, because providers practiced in California where cannabis is medically and recreationally legal, our findings may not generalize to other clinical settings. Second, the study sample was not randomly selected and may not be representative of all maternal health providers, especially due to the limited provider roles held by those who were interviewed. Third, we had limited insights from providers about caring specifically for BIPOC people, as well as the interrelationship between providing care to this group and their own identities outside of it,^46,47^ an area we wish to explore more deeply with a revised interview guide focusing more on issues of race and racism. Despite its limitations, the study includes a qualitative exploratory approach and represents provider perspectives on a taboo public health subject among providers.

Our research on provider perceptions of cannabis use and risk provides another example to consider the structural and social contexts that shape maternal health—such as individual biases and lack of expertise—that contribute to compromised care.^48^ With BIPOC and low-income people already more at risk of poor maternal health outcomes,^49^ providers may be in a position to better counsel these patients about cannabis use and work toward fostering clinical policies and practices of care which ensure that patients who use substances during pregnancy receive appropriate care.^50^

## Supplementary Material

Supplemental data

Supplemental data

Supplemental data

## Supplementary Material

Supplementary Appendix SA1

Supplementary Appendix SA2

Supplementary Appendix SA3

## Abbreviations Used

AAP: American Academy of Pediatrics
ACOG: American College of Obstetricians and Gynecologists
BIPOC: Black, Indigenous, and People of Color
DSM-5: Diagnostic and Statistical Manual of Mental Disorders, Fifth Edition
MADRES: Maternal and Developmental Risks from Environmental and Social Stressors
NIH-NIMHD: U.S. National Institute of Health, National Institute on Minority Health and Health Disparities
SBIRT: Screening, Brief Intervention, and Referral to Treatment
USC: University of Southern California
