# Supplementary material for: Legislation has Changed But Issues Remain: Provider Perceptions of Caring for People Who Use Cannabis During Pregnancy in Safety Net Health Settings, a Qualitative Pilot Study
Source: Womens Health Rep (New Rochelle). 2023 Jul 28;4(1):400–8. doi: 10.1089/whr.2023.0057 (PMC10389248; doi:10.1089/whr.2023.0057)
Supplement: Supplemental data [file Suppl_AppendixSA1.docx]

**SETUP**

- Our discussion will be about 45-60 minutes.
- E-Consent: We will be recording audio and video for documentation purposes via Zoom.
- We may interrupt you to be mindful of your time and to get to all of our questions we would appreciate your opinions on.
- My colleagues are here as well + may have some follow-up questions for you in last 15 minutes of our discussion.
- There are no right / wrong answers; we just want to get your perspective. Assume I’m naïve!
- How data used: We will talk to 10 others + record, accuracy, share w/team + thank you, importance of contribution + compensation
- Positionality: Lead/co-lead + pronouns + positionality / privilege / social positions (vs. health frontlines)

**GUIDING QUESTION**

- What themes and opportunities emerge from maternal health stakeholders sharing their experiences about caring for people who use cannabis during pregnancy?

**PURPOSE / INTENT**

- Learn about your role as a maternal health stakeholder in caring for people who use cannabis during pregnancy.
- Better understand your experiences + perspectives in managing cannabis use during pregnancy.
- Identify opportunities to improve maternal health + knowledge about use of cannabis during pregnancy for maternal health stakeholders and people who use cannabis during pregnancy.

**RESEARCH QUESTION**

- How does cannabis legalization influence maternal health stakeholders’ perceptions, beliefs, and practices about caring for people who use cannabis during pregnancy?

------------------------------------------------------------------------------- **RECORD** ---------------------------------------------------------------------

**0:00**  **(10 min) BACKGROUND: Understanding maternal health role + journey**

- **Icebreaker**: name + most proud moment in the last year as a health provider during the pandemic
- What inspired you to become a maternal health stakeholder? (probe: training, role, years in practice)
  - Patient panel: How much awareness of positive maternal health attitudes do you see among your pts? (probe: size, age, race/ethnicity, income, health concerns, etc.)
  - How do you feel things have changed since you first started here?
  - Walk thru: What does a typical prenatal visit include? (probe: exams, screenings, tests; ”problematic pt”)

VISUALIZE—where are things happening?

**0:10 (20 min)** **UNDERSTANDING CANNBIS USE + PREGNANCY: Knowledge v. perceptions v. practice**

- How does substance use come up with your patients? (probe: training in substance use, cannabis use, mental health) What do your colleagues think about substance use?
- Walk me thru: recent example when someone shared / disclosed cannabis use during pregnancy.
  - **Knowledge**: What’s been your training / education on cannabis? (probe: knowns / unknowns)
    - How do you learn about new maternal health research + guidelines + recs related to cannabis?
  - **Institution**: What are your thoughts on current cannabis research + guidelines + recs? (info sources, knowledge)
    - What policies have been implemented at your job? (e.g., tx program referral, handouts, child services)
      - What types of procedures / guidelines would you like around cannabis at your clinic?
    - Are these policies mandatory / routinely followed / case-by-case basis? How are they enforced?
    - Which policies regarding cannabis use work/not work? (probe: how seriously followed by others?)
    - How do your colleagues respond to cannabis use during pregnancy (probe: same or different?)
      - And what is your practice? How do you deal with cannabis use when it shows up with pts? (lead in to🡪practice questions)
  - **Personal**: What are your views on cannabis legalization + use during pregnancy?
  - **Practice**: What’s the current care plan for people who use cannabis during pregnancy at your job?
    - How do patients/clients respond to this?
    - What factors do you consider when assessing cannabis use with your pregnant patients/clients?
    - What data / information do you collect about cannabis from patients/clients?
    - How are patients who use cannabis monitored?
    - Who else is involved? (e.g., case worker, mental health provider, substance use provider)
    - Probe: risks, concerns; changes during perinatal period + birth; screening used w/in practice

**0:30 (20 min) EXAMINING CANNABIS USE CONSEQUENCES: Outcomes + resources +**

- What role does cannabis play in the lives of your patients/clients?
  - What are some of the reasons patients/clients describe using it? (assumption vs. discussed with them)
  - What are some of the shared characteristics among patients/clients who use cannabis during pregnancy?
- What outcomes do you observe among patients/clients as a result of cannabis use?
  - What strategies does your institution offer for managing cannabis use among pregnant pts? (e.g., therapies, medications, mental health)
  - What strategies do you use when managing cannabis use among your pregnant patients?
  - Are there specific cannabis products / use patterns that concern you among your patients?
  - How knowledgeable are your patients about cannabis use? (during pregnancy / lactation?)

**0:50 (10 min) EXPECTATIONS & WRAP UP**

- How do you interpret / discuss prenatal cannabis use with patients/clients + their families?
- What other substances do you see people use together with cannabis? (e.g., tobacco, vaping, alcohol)
- What factors impact interactions with patients/clients who use cannabis during pregnancy?
  - Probe: race/ethnicity, housing/neighborhood, age, socioeconomic status, health status
- Wishlist: What materials, training, or education would you like to have for cannabis use during pregnancy? (self, patients, practice)
- Final thoughts + questions for us? **People / colleagues you’d like us to reach out to?**

Additional questions:

**Developed by [authors] and using the following resources:**

Jarlenski M, Tarr JA, Holland CL, Farrell D, Chang JC. Pregnant Women's Access to Information About Perinatal Marijuana Use: A Qualitative Study. Womens Health Issues. 2016 Jul-Aug;26(4):452-9. doi: 10.1016/j.whi.2016.03.010. Epub 2016 May 4. PMID: 27131908; PMCID: PMC4958505.

Latuskie KA, Andrews NCZ, Motz M, Leibson T, Austin Z, Ito S, Pepler DJ. Reasons for substance use continuation and discontinuation during pregnancy: A qualitative study. Women Birth. 2019 Feb;32(1):e57-e64. doi: 10.1016/j.wombi.2018.04.001. Epub 2018 Apr 16. PMID: 29673617.
