# Supplementary material for: Legislation has Changed But Issues Remain: Provider Perceptions of Caring for People Who Use Cannabis During Pregnancy in Safety Net Health Settings, a Qualitative Pilot Study
Source: Womens Health Rep (New Rochelle). 2023 Jul 28;4(1):400–8. doi: 10.1089/whr.2023.0057 (PMC10389248; doi:10.1089/whr.2023.0057)
Supplement: Supplemental data [file Suppl_AppendixSA2.docx]

**Maternal health stakeholder study (MADRES)**

**Codebook (16 codes)**

Format:

[[**CODE NAME**]]: Definition

*Examples: data-driven instances / experiences from transcripts*

**Global codes (4)**

**Age**: discussion of age or generation in making cannabis decisions (e.g., young age of pts should not be using cannabis, older providers tend not to be ok with canna use)

**Race:** discussion of race or ethnicity in making cannabis decisions

**Class:** discussion of socioeconomic status or related class structures in making cannabis decisions (e.g., health literacy, social issues)

**Good quote:** key quote that provides insights into research questions

**Contexts influencing stakeholder perceptions surrounding patient cannabis use during pregnancy (3)**

**Changing beliefs about cannabis due to legalization**: stakeholder describing changing worldviews on cannabis as result of legalization [how legal / policy / social context influencing decisions?]

*Example: treating pts helped me learn something new about cannabis; able to have conversations about cannabis now; can talk to patients without involving CPS, “Legalizing marijuana… opens up the ability to be there for your patients, and to hear what they need”; “people are willing to share more, because they consider it legal”; “legalized here, there's a lot less stigma, “it's legal, so it's fine…not actually using drugs”*

**Perceiving struggles of BIPOC who use cannabis**: describing health experiences of BIPOC people and what they have to go through when cannabis is involved [how legal / policy / social context influencing decisions and outcomes for BIPOC patients?]

*Examples: less disposable income. less often listened to; good to legalize since BIPOC have been incarcerated for cannabis in past; not legalize, just decriminalizing it; systematic inequities*

**Lacking cannabis use information:** stakeholders lacking info on how to take action on cannabis use; unsure on how to approach cannabis in the workplace due to limited knowledge or guidelines [opposite of BASED ON PERSONAL KNOWLEDGE code] [how limited info influencing decisions?]

*Examples: vague or unclear institutional guidelines; no protocol; protocols vary by institution type; “I personally haven't had very much formal training”; “We don't really have policies with substance use”; staying up to date on research; Being open to learning about cannabis despite ambiguity with data; not great data out there, and care has to reflect what’s available; just to be on the safer side, until we get more long-term data, we just tell patients try to steer clear of it*

**Managing patient cannabis use during pregnancy (5)**

**Making decisions about cannabis use based on personal knowledge:** stakeholder taking actions or making decisions on patient’s cannabis use based on personal knowledge or feelings about cannabis (not based on medical knowledge or evidence) [how making decisions?]

*Examples: “depends on their age, it depends on who you are…politics”; informal knowledge NOT empirical knowledge*

**Building trust between health stakeholder and patient:** stakeholder trying to make pts comfortable and encourage dialogue surrounding cannabis use during pregnancy; trying to create a safe space and build rapport for patients who use cannabis [distinct from DISCLOUSRE] [how negotiating these decisions?]

*Examples*: *trying to avoid judgment, aim for respect; “we also shouldn't be exposing moms to stigma and potential discrimination”; “desire to build a therapeutic relationship, and not make people feel judged”; “we don’t want to isolate them”; “we just treat her like everybody else”, “really having the time to really have a patient open up to you, to establish trust”; not probe further, leave door open to talk about*

**Normalizing patient cannabis use**: stakeholders reasoning for accepting / rejecting cannabis use; describing alternatives to cannabis use or less risky behaviors [what decision are they making?]

*Examples: fine to help manage nausea; if going to use—do topical, not inhale, providing alternatives to cannabis use; taking a harm-reduction approach to managing cannabis use*

**Considering legal risk of disclosure**: how navigating legal implications of disclosing cannabis use, describing actions and reporting of use [how manage legal implications?]

*Examples: Nervous about disclosing, triggered by past interactions with social work, open DCFS cases; “patient can just not say that they use it and then they just don't get that pushback;” “hard to do effective and efficient screening, bc pts and providers are so scared that the system's gonna come and make it worse”*

**Considering medical risk of disclosure and subsequent consequences:** how navigating medical implications of disclosing cannabis use; describing the next steps regarding interventions [how manage medical implications?]

*Examples: screening and subsequent care process; cannabis on postpartum and not knowing what to do*

**Perceiving patient cannabis use (2)**

**Explaining patient motivations for cannabis use:** describing why a patient would use cannabis to relieve/treat pregnancy (e.g., physical and mental health); understanding how cannabis function in pt life

*Examples: help with the side effects of early pregnancy; “anxiety, stress relief, and nausea;” depression; “coping mechanisms and one of those might be cannabis use;” hyperemesis; self-medicating*

**Comparing cannabis to other substances:** drawing comparisons of cannabis to other substances (including pain meds)

*Examples: meth use; “It's not a huge addiction, like methamphetamine or heroin is. Why can't you just stop?; “Like you shouldn't do it in pregnancy, but they're not gonna treat them like a drug addict”; co-use; “in their minds, it would be better than using opioids”*

**Providing and outsourcing patient care (2)**

**Wanting resources**: describing resources for stakeholder / pts that stakeholders would like to manage cannabis use during pregnancy; also lacking resources

*Examples: handouts not helpful, group classes, brief summary of law, some info on implications of cannabis use to empower pts; mental health clinic; time limitations*

**Involving others in care of patients who use cannabis:** describing collaborating with multiple people (family, diff staff, departments, experts) to care for patient who use cannabis

*Example = if partner using it, hard to get pt to stop; needing multidisciplinary team to respond to use (mental health, co-use), took more than one provider to get health they need, interdependence on others; bringing in social worker; system pipelines that work or don’t; “Getting our pediatric people on board for long-term effects. Things that we don't see once they leave the hospital.”; “if it's not symptom-related, and it's just substance use related, then I think you have to get other people involved”*
